# The fast-acting “pulse” of Heinrich Stadial 3 in a mid-latitude boreal ecosystem

**DOI:** 10.1038/s41598-020-74905-0

**Published:** 2020-10-22

**Authors:** Federica Badino, Roberta Pini, Paolo Bertuletti, Cesare Ravazzi, Barbara Delmonte, Giovanni Monegato, Paula Reimer, Francesca Vallé, Simona Arrighi, Eugenio Bortolini, Carla Figus, Federico Lugli, Valter Maggi, Giulia Marciani, Davide Margaritora, Gregorio Oxilia, Matteo Romandini, Sara Silvestrini, Stefano Benazzi

**Affiliations:** 1 Department of Cultural Heritage, University of Bologna, 48121 Ravenna, Italy; 2 Research Group on Vegetation, Climate and Human Stratigraphy, Laboratory of Palynology and Palaeoecology, CNR-Institute of Environmental Geology and Geoengineering (IGAG), 20126 Milan, Italy; 3 Department of Environmental and Earth Sciences, University of Milano-Bicocca, 20126 Milan, Italy; 4 CNR-Institute of Geosciences and Earth Resources (IGG), 35131 Padua, Italy; 5 School of Natural and Built Environment, Queen’s University Belfast, Belfast, BT7 1NN UK; 6 Dipartimento di Scienze Fisiche, della Terra e dell’Ambiente, Università di Siena, 53100 Siena, Italy; 7 Dipartimento di Scienze Chimiche e Geologiche, Università di Modena e Reggio Emilia, 41125 Modena, Italy; 8 Dipartimento di Studi Umanistici, Sezione di Scienze Preistoriche e Antropologiche, Università di Ferrara, 44100 Ferrara, Italy; 9 Department of Human Evolution Max Planck Institute for Evolutionary Anthropology, 04103 Leipzig, Germany

**Keywords:** Climate sciences, Ecology, Environmental sciences

## Abstract

A 3800 year-long radiocarbon-dated and highly-resolved palaeoecological record from Lake Fimon (N-Italy) served to investigate the effects of potential teleconnections between North Atlantic and mid-to-low latitudes at the transition from Marine Isotope Stage (MIS) 3 to 2. Boreal ecosystems documented in the Fimon record reacted in a sensitive way to millennial and sub-millennial scale Northern Hemisphere atmospheric circulation patterns. The high median time-resolution of 58 years allows the identification of five abrupt event-boundaries (i.e., main forest expansion and decline excursions) synchronous with the sharp stadial/interstadial (GS/GI) transitions within dating uncertainties. During Heinrich Stadial 3 (HS 3) we reconstruct more open and dry conditions, compared to the other GS, with a dominant regional scale fire signal. Linkages between local fires and climate-driven fuel changes resulted in high-magnitude fire peaks close to GI/GS boundaries, even exacerbated by local peatland conditions. Finally, palaeoecological data from the HS 3 interval unveiled an internal variability suggesting a peak between 30,425 and 29,772 cal BP (2σ error) which matches more depleted δ^18^O values in alpine speleothems. We hypothesise that this signal, broadly resembling that of other mid-latitudes proxies, may be attributed to the southward shift of the Northern Hemisphere storm tracks and the associated delayed iceberg discharge events as documented during other HS.

## Introduction

Abrupt climate changes, in particular Heinrich Stadials (HS, sensu Sanchez Goñi and Harrison^1^) and Dansgaard-Oeschger (D-O) cycles (i.e. Greenland Stadial/Interstadial transitions or GS/GI^2^), were active during Pleistocene glacial cycles^3,4^ and particularly well documented, in terms of structure and timing, during the last glacial cycle^2,5^.

Model simulations show that a rapid (i.e. within few years) transmission of such abrupt ‘‘flips’’ occurred through a latitudinal displacement of the Intertropical Convergence Zone (ITCZ) over the Atlantic Ocean and its margins^6^. Changes in ocean heat transport were accompanied by rapid reorganizations in atmospheric circulation^7^, probably lagging by a few years only^2^, and dust regime shifts at stadial/interstadial boundaries^8^. Greenland dust mostly sourced from central Asian deserts^9–11^ points to a large scale atmospheric signal probably associated with intensified southward shifts of the ITCZ and strengthening of the westerlies, particularly during HS^12,13^.

The effects of HS, spread across much of the Northern Hemisphere, were identified as cold episodes in marine records^14–16^, cold and dry phases in European and Asian speleothems^17–20^, terrestrial palaeoecological records^21–23^, and expressed in the loess stratigraphic successions^24^. Also, the fine linkage of these millennial-scale oscillations to the Indo-Asian monsoon system is proven in a number of palaeoclimate records of the Afro-Asian realm^25,26^ and the western (tropical) Atlantic, although with variable responses to HS phases^27,28^.

Limitations in establishing whether these events are synchronous at different latitudes and/or ecogeographic zones are (1) the often inadequate temporal resolution of proxy records and (2) the relatively large chronological uncertainties associated with short transitions (on centennial and sub-centennial timescales)^29,30^. Where possible, independent chronological information (i.e. the identification of known tephra/cryptotephra layers) may provide additional support for the validity of alignment approaches^31^.

Cave stalagmite δ^18^O records with radiometric U-Th dating are a notable exception being among the most accurately datable archives (i.e. within the last ca 100 ka BP, two sigma (95%) errors can be below 1% of the U-Th age) and considered excellent archives for recording short-term climate fluctuations^17,18,20,32,33^. Unfortunately, their registration is often fragmentary, as hiatuses may occur during cold/dry phases (i.e. HS 5 and HS 4)^19^ and the response to climatic conditions can be influenced by regional and site-specific factors^34,35^.

These issues can be overcome by continuous palaeoecological series that are stratigraphically well constrained through high- (at least sub-millennial) resolution^21^. Palaeoecological records take advantage of joint analyses of different proxies which allow to explore the complex relationships occurring between temperature changes (as recorded in oxygen isotopes) and terrestrial ecosystems. To Investigate such relationships, multivariate analysis can help to extract major structures in the data (i.e., ecoclimatic gradients) and to identify target pollen descriptors of past climate changes. Moreover, abrupt climate changes may affect all the levels of biological plants response: i.e. quantitative changes in pollen production represent the fastest response to climatic change, within a year or two^36^. Finally, additional information about local and regional fire signals may be profitably used to further decipher the climate fingerprint, although they may cause disproportionate effects on vegetation structure^37^.

### Placing Heinrich Stadial 3 in the framework of mid-latitude climate dynamics

The cold intervals associated with HS in the Atlantic records^38^ (Fig. S1) were recently recognized in GISP2, GRIP and NGRIP δ^18^O ice cores and unambiguously identified during GS^2,39,40^. Here, we focus on HS 3, which corresponds to GS 5.1 (30.6–28.9 b2k)^2,5,41,42^, although it is less expressed in the Greenland δ^18^O record if compared to other Heinrich Stadials. HS 3 is part of the complex sub-millennial dynamics which led to the maximum expansion of European mountain glaciers around 26 cal kBP^43–45^ and of the British Islands Ice Sheet^46^.

We investigated such dynamics through palaeoecological, geochemical and geochronological analysis of the Lake Fimon record at the MIS 3–2 transition. The geographic position of the site at the south-eastern fringe of the Italian Alps is particularly suitable for capturing the regional effects of climate changes on both vegetation and alpine glaciers^47,48^. Radiocarbon dating provides an independent (i.e. non-tuned) means of age control for the high-resolution pollen-based event-stratigraphy.

**Figure 1:**
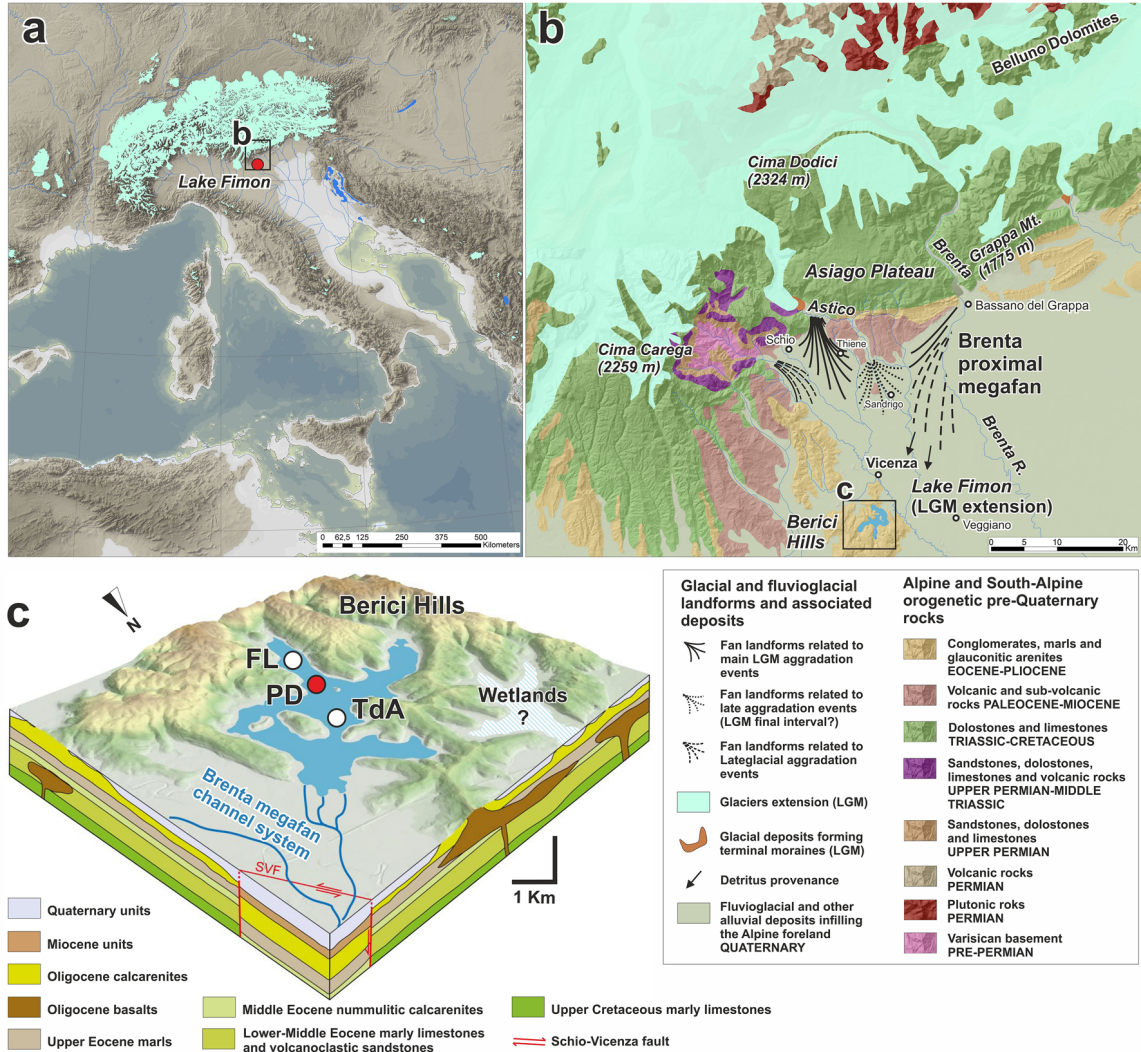
(**a**) Palaeogeographic map of the Central Mediterranean, the Alpine region, the Italian Peninsula and Western Balkan Peninsula during the Last Glacial Maximum. Digital Elevation Model (DTM: 25 m cell size, source: https://land.copernicus.eu/imagery-in-situ/eu-dem/eu-dem-v1.1). Sea level drop at − 130 m^56^. Alpine glaciers downloaded from https://booksite.elsevier.com/9780444534477/ and modified on the Italian side according to updated reconstructions^43,57–61^. Adriatic lakes^62^ and rivers simplified after Maselli et al.^63^. (**b**) Geological and glaciological sketch of the study area showing the LGM and Lateglacial evolution of the Veneto Prealps. The geological map is based on the “Structural model of Italy”^64^ and local geological maps^57,65–69^. Geological formations are superimposed on a digital elevation model for the eastern Southalpine chain (DTM; 10 m cell size, source: https://www.regione.veneto.it/web/agricoltura-e-foreste/modello-digitale-del-terreno) merged with an elevation model for its foreland basin (DTM; 25 m cell size). (**c**) 3D view (DTM; 5 m cell size) of the northern area of the Berici Hills hosting Lake Fimon (here at its LGM maximum extent), geological reconstruction of the subsoil after^70–73^. The location of Fimon cores drilled in the Fimon basin are also indicated: Ponte sulla Debba (PD), Torri di Arcugnano (TdA) and Fimon Lago (FL). We produced the maps in this figure using Esri’s ArcGIS 10.7 software (https://www.esri.com/software/arcgis).

The studied deposit developed in the context of a large palustrine basin built-in an articulated valley system (Fig. 1) not perturbated by glacier outbursts, allowing to obtain a continuous sub-millennial to sub-centennial registration. The record is exceptional in that stable deposition occurred in a peat system similar to present-day boreal zones of West Siberia, northern Europe and Canada where the degree of paludification reaches 50% and more^49,50^. Very few other palaeoecological records exhibit a comparable resolution during MIS 3–2 (Monticchio, Megali Limni, Tenaghi Philippon^51–55^). Among these, although in the context of the Mediterranean climate region, the Tenaghi Philippon site also shares similar depositional conditions being an extensive intramontane paludified area, highlighting the potential of such settings in capturing the response of plant ecosystems even during abrupt climatic shifts.

Our research takes advantage of updated stratigraphic records at the southern side of the Alps (Fig. 1). Here correlations between glaciers’ spread^43,57^, aggradation of the outwash plain^74^ and lake formation/evolution^48^ allowed robust paleolandscape interpretation (Fig. 1b). Data on the detritus provenance^48,57^ helped with reconstructing the interplay between the evolution of the plain and of Lake Fimon sedimentary succession (Fig. 1c and see SI-2).

## Results

### Fimon palynostratigraphic record

**Figure 2:**
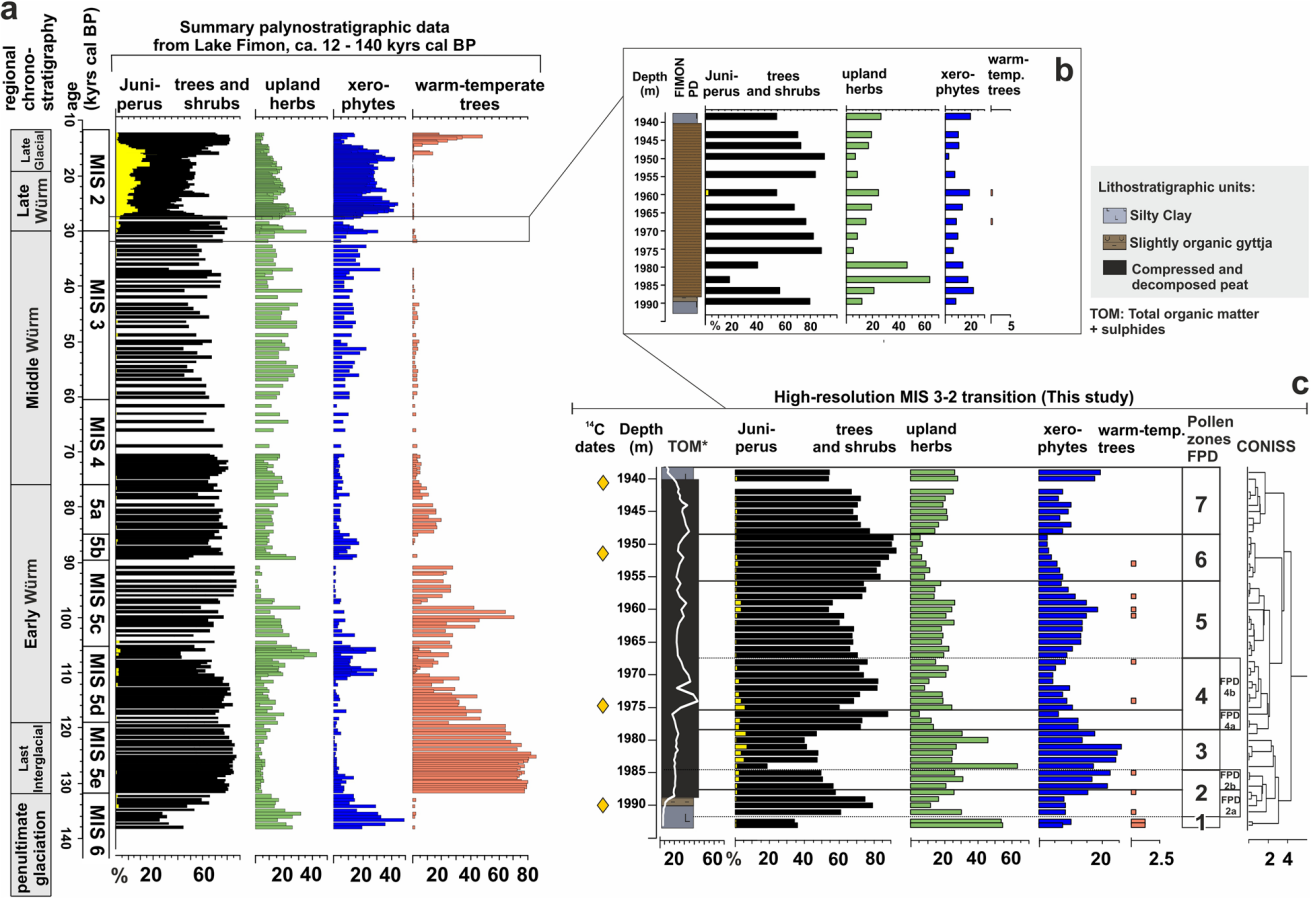
Summary palynostratigraphic data from Lake Fimon. (**a**): synthetic composite pollen record obtained from cores FL (12–27 ka) and Fimon PD (> 27 ka) and documenting the history of plant ecosystems during the whole Late Pleistocene (modified after Monegato et al.^48^). Only selected pollen curves are shown: sum of trees and shrubs (black), *Juniperus* (yellow), upland herbs (light green), xerophytes (blue): sum of *Artemisia*, Chenopodiaceae, *Helianthemum*, Ephedraceae, *Centaurea scabiosa*, warm-temperate woody plants (pink): sum of *Corylus*, deciduous *Quercus*, *Tilia*, *Ulmus*. (**b**) Selected interval indicated by a rectangle and analysed at low resolution in a previous study^47^. (**c**) New high-resolution paleoecological record from the Fimon PD core, with special attention to the MIS 3–2 transition.

The long lacustrine-palustrine succession of Lake Fimon provided a continuous record of terrestrial ecosystems covering the complete Late Pleistocene^47,48^ (Fig. 2). In order to investigate in detail the effects of abrupt climate variability across the MIS 3–2 transition, a total of 54 samples were analysed for pollen (Fig. 2c), throughout a peaty-gyttja and clay interval allowing for a stratigraphic resolution of 1 cm per sample between 19.39 and 19.93 m depth (see SI-2 and Fig. S2). On a long-term scale, the reduction of warm-temperate elements (deciduous *Quercus* and other thermophilous taxa) in favour of pine woodlands, suggests a shift towards colder conditions. Pollen data pinpoint the recurrent pattern of forest fluctuations dominated by *Pinus sylvestris/mugo* with other trees and shrubs (values shifting between 18 and 93%) characterising the boreal biome^75,76^. Forest withdrawals are centred in the FPD2b-3, FPD5 and FPD7 pollen-zones. A major shrinking of the forest patches was evidenced in FPD2b-3 pollen-zones by the substantial decrease in *Pinus sylvestris/mugo* percentages (from 75 to 10%) mirrored by the expansion of xerophytes (e.g. *Artemisia* and Chenopodiaceae) and upland herbs up to 25% and 64% respectively (Fig. 2c).

### Chronology

**Figure 3:**
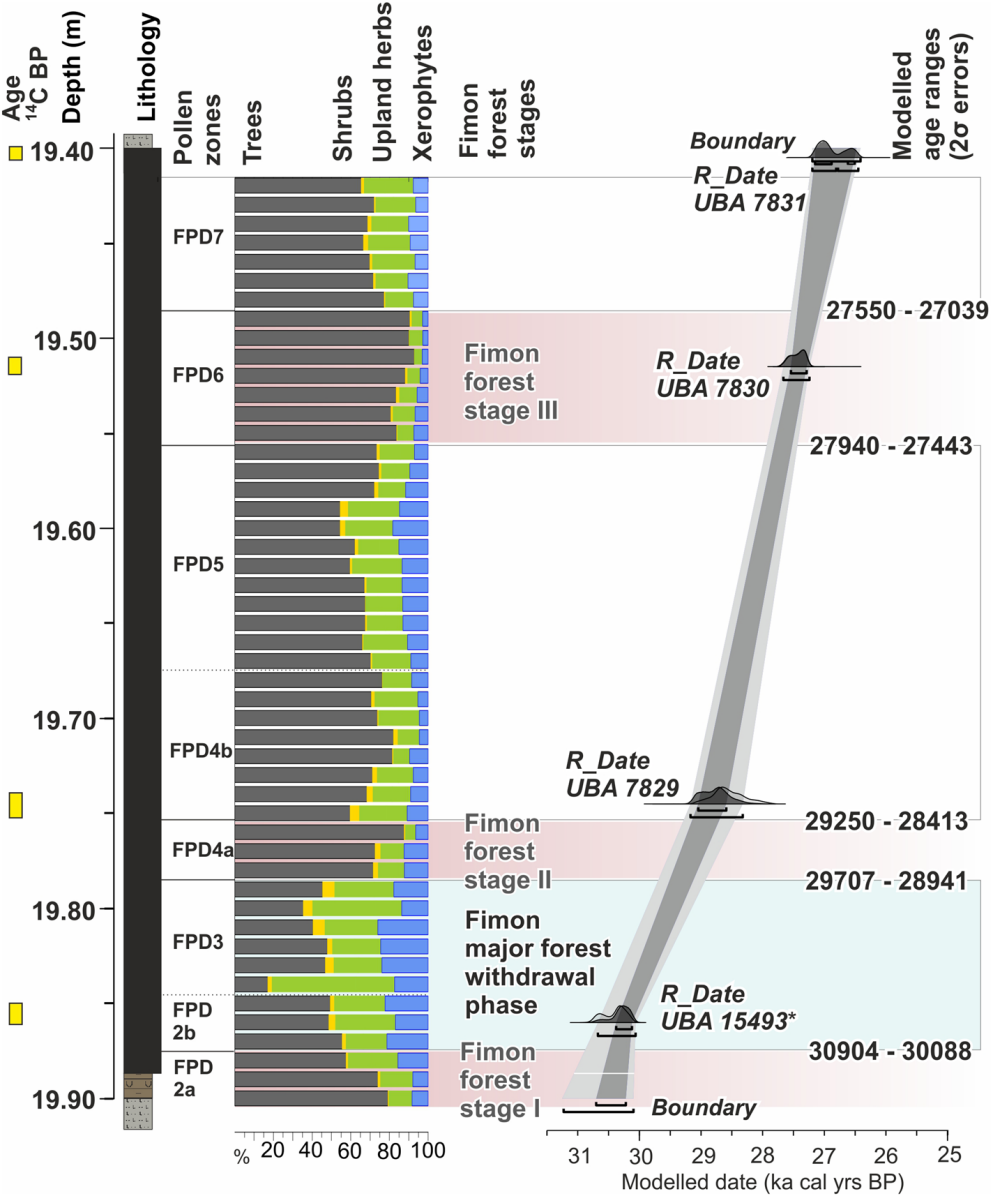
Age-depth model of 19.90–19.405 m Fimon PD interval calculated with the OxCal 4.4 calibration software^77^ using IntCal20 calibration curve^78^. The pollen zonation and the modelled mean ages at the pollen zones boundaries, defining the Fimon forest stages I–II–III, are also shown.

We modelled data from the peaty-gyttja interval (19.90–19.405 m, LZ1-2—see Fig. 3). The list of radiocarbon ages included in the age-depth model (see Fig. S3 and “Methods” section for further details) is shown in Table 1. These organic deposits span ca. 3800 years over 49 cm, i.e. between 30.6 and 26.8 ka cal BP (Fig. 3). The median resolution for pollen samples is 58 years, whereas the median resolution for macrocharcoal samples is 30 years. Overall, steady pollen percentages variations and homogeneous depositional conditions suggest a continuous undisturbed accumulation with no evidence of erosional surfaces (median accumulation rates = 0.01 cm/years). Five abrupt (multidecadal to centennial scale) event-boundaries, defined by pollen zonation (Figs. 2, 3), are constrained at 30,904–30,088 (end of forest stage I), 29,707–28,941 (start of forest stage II), 29,250–28,413 (end of forest stage II), 27,940–27,443 (start of forest stage III) and 27,550–27,039 (end of forest stage III) cal BP (2σ error) (Fig. 3).

**Table 1:** List of Fimon PD and TdA radiocarbon ages.

Radiocarbon ages from Lake Fimon—Ponte sulla debba (PD) and Torri di Arcugnano (TdA) cores							
Lab code	Core	Lithology	Depth (m)	Material dated	^14^C Age BP	2σ calibration range (cal years BP) IntCal20	Median probability (cal years BP)
UBA-7831	Fimon PD	Peat	19.40–19.41	Bulk sediment	22,593 ± 115	26,454–27,213	26,954
UBA-7830	Fimon PD	Peat	19.51–19.52	Bulk sediment	23,165 ± 116	27,252–27,683	27,438
UBA-7829	Fimon PD	Peat	19.74–19.755	Bulk sediment	24,376 ± 187	28,037–29,058	28,596
UBA-15493	Fimon TdA	Peat	19.85–19.86	Bulk sediment	26,158 ± 97	30,125–30,768	30,376
UBA-7828	Fimon PD	Slightly organic gyttja/silty clay	19.89–19.91	Bulk sediment	25,476 ± 114^a^	29,322–30,027	29,797
UBA-17120	Fimon PD	Slightly organic gyttja	20.24–20.26	Pollen concentrates (15 µm < fraction < 41 µm)	23,206 ± 95^a^	27,279–27,687	27,465
UBA-17121		Slightly organic gyttja		Pollen concentrates (41 µm < fraction < 90 µm)	21,379 ± 122^a^	25,516–25,937	25,741
UBA-17122		Slightly organic gyttja		Pollen concentrates (90 µm < fraction < 250 µm) (charcoal enriched fraction)	19,972 ± 70^a^	23,819–24,188	23,981
UBA-17123		Slightly organic gyttja		Bulk sediment	23,606 ± 86^a^	27,630–27,906	27,765
UBA-17118	Fimon PD	Slightly organic gyttja	20.36–20.38	Pollen concentrates (41 µm < fraction < 90 µm)	22,035 ± 107^a^	25,947–26,476	26,224
UBA-17117		Slightly organic gyttja		Pollen concentrates (fraction > 90 µm) (charcoal enriched fraction)	22,424 ± 113^a^	26,403–27,064	26,742
UBA-17119		Slightly organic gyttja		Bulk sediment	26,566 ± 133^a^	30,427–31,086	30,873

^a^Indicates the^14^C date excluded from the final age-depth model solution. For those ages already published in Pini et al.^47^ the uncertainty has been increased retrospectively to account for long term variability in the background.

### Leading ecological gradients under GI-GS variability

**Figure 4:**
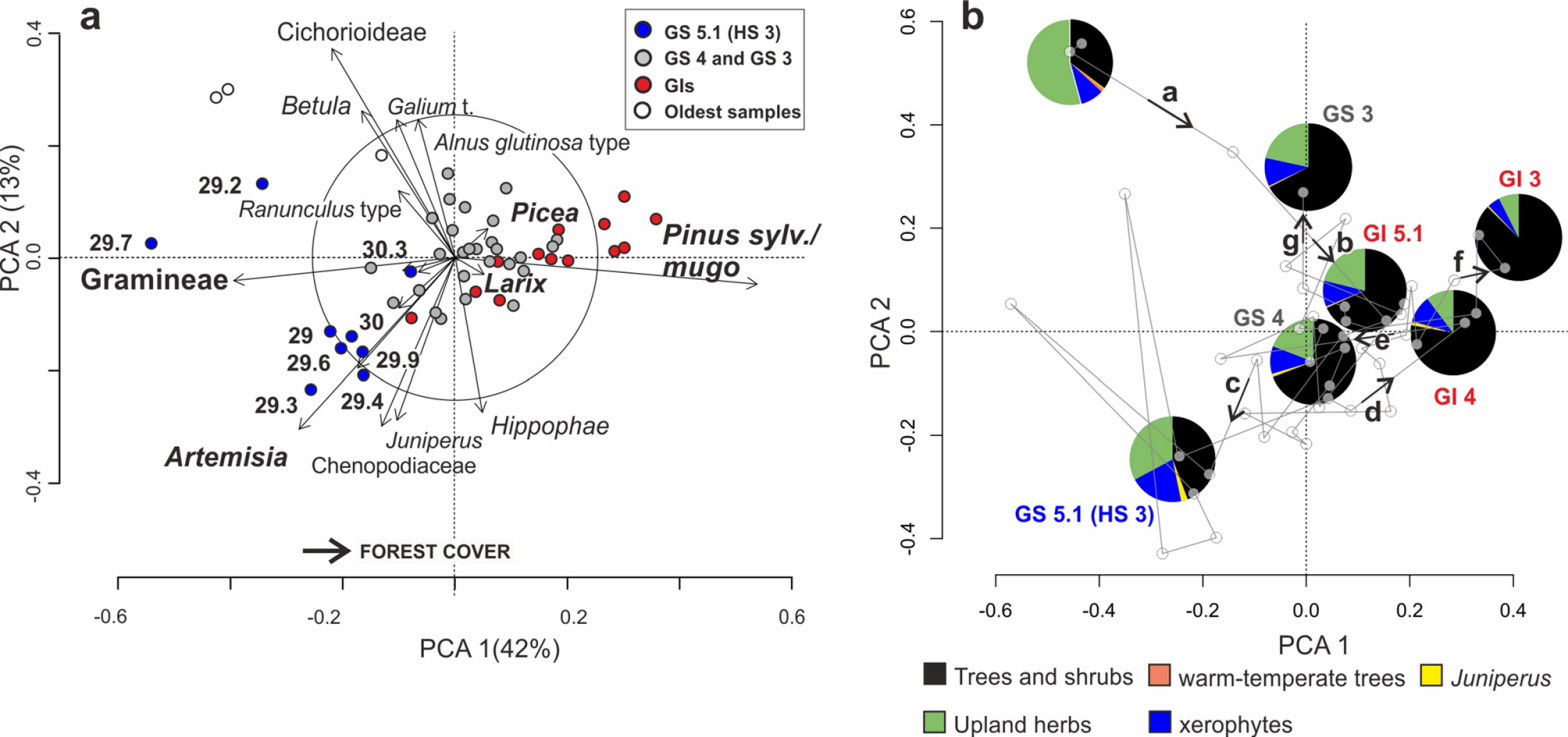
(**a**) PCA ordination of terrestrial pollen taxa (> 2%) and sites showing changes in the pollen image for terrestrial ecosystems structure. Axis 1 represents a forest cover gradient. (**b**) PCA ordination of sites. Pie-charts show the relative abundances of different vegetation types at GS and GI intervals. Data standardization and ordination were carried out with the Vegan package^79^ in R environment^80^.

The ordination analysis of terrestrial pollen data (Fig. 4) shows that most of the variance (42%, PCA axis 1) is related to a forest cover gradient where *Pinus sylvestris/mugo,* together with *Larix and Picea* (positive scores) contrast Gramineae (negative scores). The second axis (13.2% of the total variance) opposes lakeside forests (i.e. *Betula* and *Alnus glutinosa*) and upland herbs (Cichorioideae and *Galium*) to xerophytic taxa (Fig. 4a). Data from FPD 2b-3 pollen zone (blue dots, Fig. 4a) display the most negative values of the sequence and are positioned in the lower left part of the biplot indicating extremely open condition in contrast to FPD 2a-4a and 6 pollen zones (red dots, Fig. 4a), identifying Fimon forest stages I, II and III (Figs. 3 and 4b).

### Macrocharcoal analysis and local fire reconstruction

**Figure 5:**
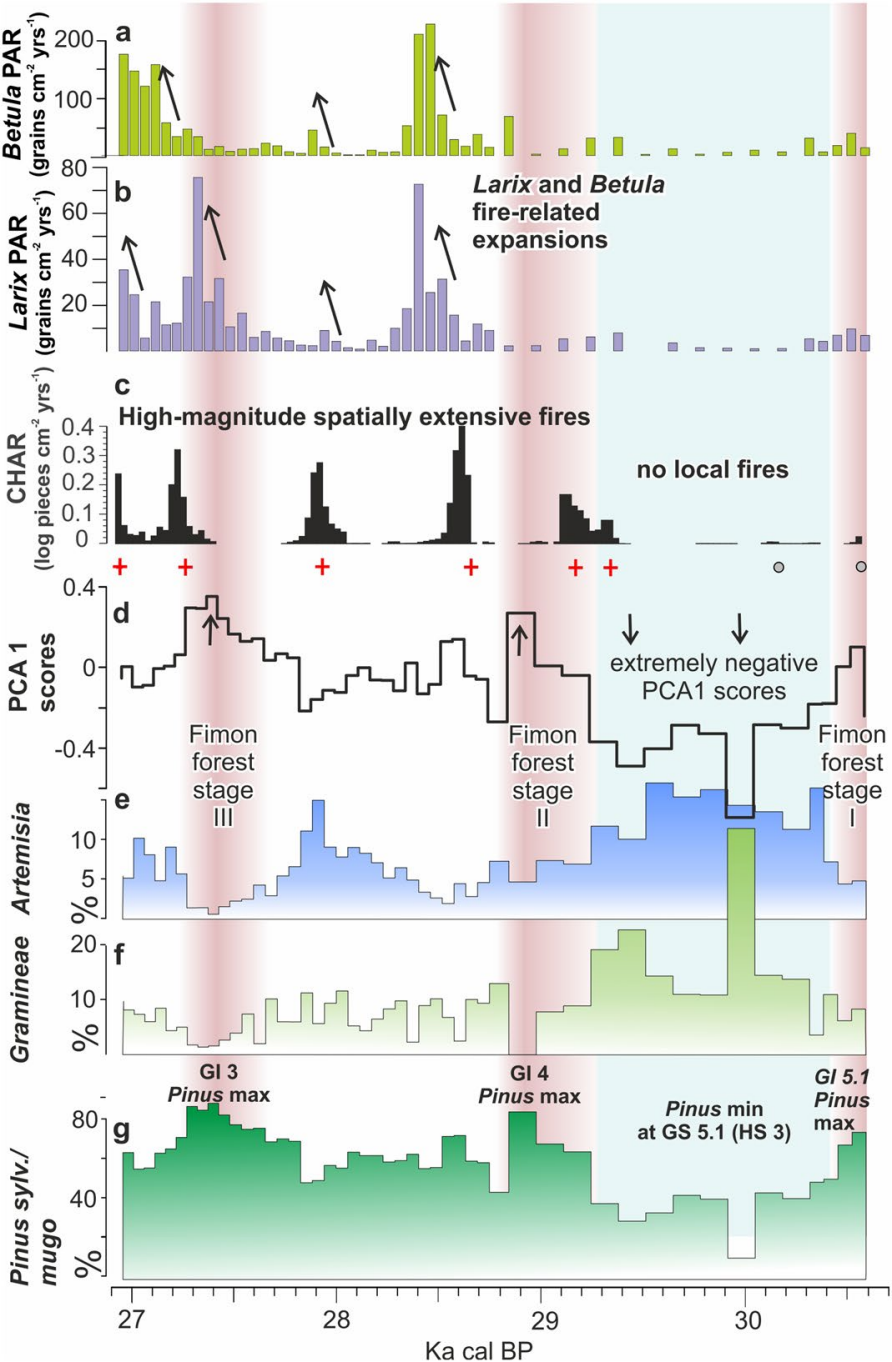
Summary plot showing Fimon PD palaeoecological data on time: (**a**) *Betula* pollen accumulation rate (PAR); (**b**) *Larix* and *Betula* pollen accumulation rates (PAR); (**c**) Macroscopic charcoal (> 125 µm sized) accumulation rates (black histograms). Local fires identified in the Lake Fimon record are indicated by a red cross or grey dots depending on their status (positive or negative passing the charcoal peak screening, respectively) using the method implemented in CharAnalysis 0.9 software^81^; (**d**) PCA 1 scores indicating forest-cover gradient; (**e**) *Artemisia* % curve; (**f**) Gramineae % curve; (**g**) *Pinus sylvestris/mugo* % curve.

We investigated changes in fire activity through the analysis of sieved charcoal particles in the analysed core section. Concentrations of sieved charcoal fractions (i.e. 62–125 µm, 125–500 µm and > 500 µm, Fig. S5) were found to be similar, suggesting comparable trends in fire dynamics at local to extra-local scales. To investigate local fires, influx of charcoal particles > 125 µm has been taken as a proxy of fire history within a few kilometres from the study area (see “Methods”). Most of the record (87%) has a signal to noise index greater than the critical value of 3 (median = 5.05, min = 0 and max = 7.5; Fig. S6), as determined by Kelly's method, and can be considered appropriate for peak detection. Peak analysis revealed six statistically significant CHAR peaks between 30.6 and 26.8 cal kBP showing moderate-to-high magnitude (between 0.2 and 2.3 pieces cm^−2^ peak^−1^) and a relatively stable 400-year mean Fire Return Intervals (mFRI). No local fires occurred between 30.6 and 29.4 cal kBP (Fig. 5).

## Discussion

### Timing of ecosystems response to GI-GS variability

The glaciated Alps represented an effective physiographic barrier for meridional advection and caused increase of humidity in the south-eastern alpine region^32,82^ hosting Lake Fimon. Because of this rainfall boundary, windward southern Alps maintained almost persistently forested environments during the last 140 years cal BP^47^. The established configuration favoured very little migrational lags because conifers and cold-resistant deciduous species persisted in the region and rapidly responded to abrupt climate changes. Other studies suggest that vegetation response to North Atlantic climate change was rapid and effectively synchronous across southern Europe^36,83,84^ with Greenland climate variability within dating uncertainties.

**Figure 6:**
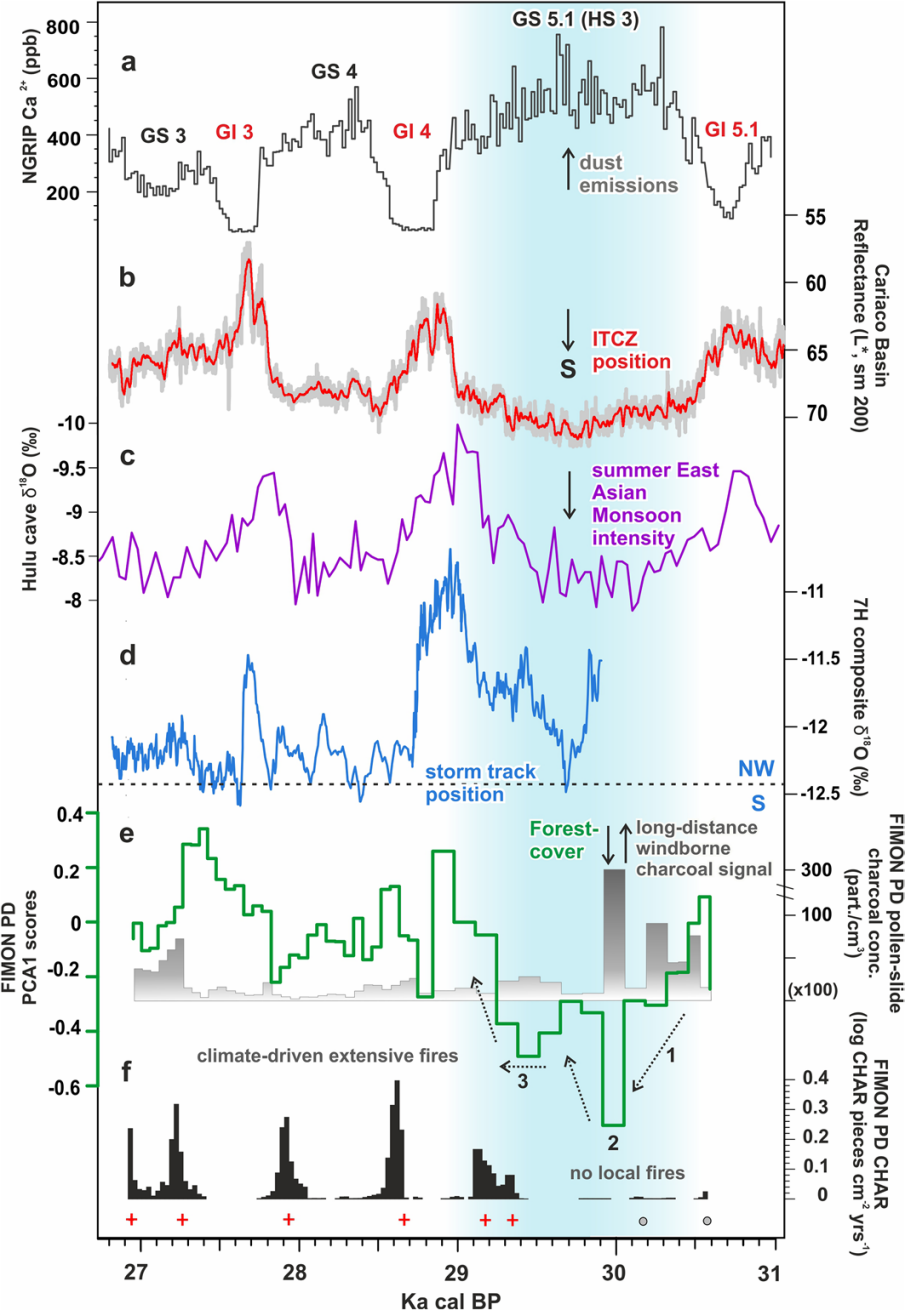
Selected series of climate proxies plotted according to their calendar chronology and compared with the proxy series obtained in Fimon PD record. Key to panels: (**a**) NGRIP dust (Ca^2+^) record on the GICC05 chronology^2^; (**b**) Cariaco Basin MD03-2621 L* record on IntCal13 radiocarbon chronology fine-tuned to the NGRIP δ^18^O GICC05 age scale (red, 100-point running mean)^27^; (**c**) Hulu cave δ^18^O record on the U–Th based chronology^86^; (**d**) 7H stalagmite δ^18^O record on the U–Th based chronology^32^; (**e**) Fimon PD PCA 1 scores and microcharcoal (10–50 μm sized) concentration records, indicating respectively forest-cover gradient and long-distance windborne charcoal signal, on Fimon radiocarbon based chronology; (**f**) Fimon PD macroscopic charcoal (> 125 μm sized) accumulation rates indicating recurrent climate-driven extensive fires, plotted on Fimon radiocarbon based chronology. Local fire peaks are indicated by red crosses. Light blue area indicates GS 5.1 (HS 3) interval. See “Discussion” for more details about 1–2–3 phases.

The main pollen descriptors in Fimon PD record (i.e., *Pinus sylvestris/mugo*, *Artemisia*, *Gramineae*, Fig. 5e–g) show rapid responses in terms of quantitative changes in pollen production within few decades. The high median time-resolution of 58 years allows the identification of five abrupt event-boundaries (i.e., main forest expansion and decline excursions). Such events are synchronous with the sharp GS 5.1 (HS 3), GI 4, GS 4, GI 3 and GS3 starts in the NGRIP δ^18^O (GICC05 chronology) within dating uncertainties (Table S1). Between 30,904 and 30,088 (end of Fimon forest stage I) and 29,707–28,941 (start of Fimon forest stage II) cal BP (2σ errors) a millennial phase of major ecosystem transformation linked to GS 5.1 (HS 3) interval is documented (see the next section for further details). Our basal age for this interval also fits the modelled age of 29,675–30,964 cal BP (2σ error) from the site of Casaletto Ceredano^85^, indicating a significant increase in continentality in northern Italy related to a lockdown of moist westerlies intervening with the onset of HS 3^85^. Despite a general consistency between NGRIP and Fimon PD event stratigraphies, we observed a weaker signal agreement between 28.5 and 29.5 cal kBP. The end of HS 3 interval (i.e., marked by the onset of GI 4), is 460 years younger in GICC05 chronology as compared to Fimon PD record, although age offsets remain numerically within uncertainties (Table S1). Similar discrepancies are also observed between NGRIP and other independent chronologies of speleothem isotopic records at GI 4 start (i.e. Sofular, 7H and Hulu cave)^17,32,86^: ∆_t NGRIP-Sof_ = − 550 years, ∆_t NGRIP-7H_ = − 209 years and ∆_t NGRIP-HULU_ = − 497 yrs (Table S1 and Fig. 6). These data point to an ice core chronology generally younger during this time interval as already outlined by Fleitmann et al.^17^.

In this framework, the Lake Fimon record shows evidence for a rapid and sensitive ecoclimatic response to abrupt stadial/interstadial climate changes, offering the opportunity to compare ice, marine and mid-latitude terrestrial records across the MIS 3–2 transition.

### Terrestrial ecosystems structure during Heinrich Stadial 3

At the onset of HS 3, boreal forest ecotypes were largely replaced by open environments at the continental edge of the forest-steppe ecological gradient (i.e., end of Fimon forest stage I, Figs. 3 and 4). Herbaceous communities represented by sedges and grasses expanded together with xerophytic woody perennials and forbs with *Artemisia* and Chenopodiaceae species. Pollen input from trees was mostly due to large pollen producers such as *Pinus sylvestris/mugo* and *Betula*, likely forming small pine-birch groves in forest-steppe zones. *Juniperus* underlines the openness in the canopy (Fig. S4). The occurrence of scattered trees of spruce and larch may also be inferred, while pollen of all warm-temperate woody plants was completely absent, apart from sporadic *Corylus* and *Alnus glutinosa* type (Fig. 2 and Fig. S4).

During HS 3 [between 30,904 and 30,088 (end of Fimon forest stage I) and 29,707–28,941 (start of Fimon forest stage II) cal BP (2σ errors)] *Artemisia*-dominated semideserts, steppe and meadow-steppe, including Gramineae and Asteroideae, occupied large areas. Boreal forests were possibly close to the so-called continental timberline limit^87^.

Modern pollen analogues for these vegetation communities can be found in the intermountain systems of the Altaj-Sayan-Mongolian border (Fig. S7)^88–90^ under climates characterized by cold winters (mean January T between − 20 and − 25 °C), mean July temperature around 15 °C and mean annual precipitation at ca 300 mm^91,92^. Under the continental climate of the Mongolian region, the effective humidity, resulting from the balance between evapotranspiration and precipitation, largely controls the environmental conditions^93^. Here, *Pinus sylvestris* forests are mostly found on dry south-facing slopes in the more oceanic northern parts of the Western Sayan. This species is more thermophilous (optima around 15–16 °C, mean July T) and moisture demanding (optima around 700–900 mm) than *Larix sibirica* and *Pinus sibirica*^89^. Similarly, the reconstructed Fimon PD gradient (i.e., PCA 1 scores, Fig. 5d) may have been driven by moisture availability together with temperature, acting as limiting factor for tree growth especially during HS 3 (Fig. 5).

Although with a coarser resolution, similar changes were recorded during HS, and in particular during HS 3, in different sites north of the Alps (i.e. La Grande Pile, Les Echets, Bergsee)^23,94–96^. In the Mediterranean area, hydrological and thermal stresses associated with HS generally resulted in large contractions of forests, with an almost complete depletion in forest cover (i.e. Tenaghi Philippon, Megali Limni, Kopais)^52,97–99^. Differently, in sites where moisture availability was not a limiting factor (i.e. Ioannina^84^), differences between GS and HS magnitude of climate forcing seem to be better expressed^22^.

### Influence of sub-millennial climate oscillations on fire-regime and local signal amplification

Fimon boreal ecosystems experienced low fire-frequency (mean value = 1.6 fires 1000 years^−1^) with a stable mean fire return interval of about 400 years (Fig. S6). During HS 3 interval, besides climate conditions being dry enough to promote fires, it is very likely that the high degree of openness resulted in very limited to no local fuel availability for fire to spread (Fig. 5c). Our results seem to be in agreement with a study from comparable Norwegian landscapes^100^ showing fire return intervals of 288–1141 years, also corroborated by other evidence from the Eurasian taiga zone, where mean fire return intervals are significantly longer in peatland boreal environments than in upland systems^101–103^.

Despite warm conditions being expected to increase the size and frequency of fires in boreal biomes^104,105^, Fimon record shows no fire activity in phase with peaks of maximum tree populations expansion suggesting an important role of moisture as limiting factor during forest stages (Fig. 5). High-magnitude fire peaks occurred (or started) during stadials close to GI/GS boundaries when biomass availability was higher than during more open stadial conditions, water table was lower, and prolonged seasonal frozen ground (or permafrost patches enlargement) may have induced drier conditions at the peatland interface. Such conditions arguably enhanced fuel consumption per fire episode favouring extensive fires across lowlands and the uplands surrounding the peatland, exacerbating the effects of climate forcing on fire regime^106^. Similar conditions can be sought in modern Canadian or Siberian transitional forest-steppe ecotones, where fires can spread freely over whole watersheds after summer rainless periods characterized by lightning storms^76,107^. At Fimon, flammable species adapted to wildfires (i.e. *Larix, Betula*) appear to benefit from local fires, at least indirectly, showing post-fire near-exponential population growth (Fig. 5a,b). *Larix* specimens were able to colonize peatlands, even during periods of extended frozen season (GS), as they can inhabit permafrost soil^108^ because of the phenology of needle-fall^109^, their enhanced nitrogen allocation after fire events^110,111^ and their high tolerance to the freezing stress of the winters^112^. Remarkably, the recovery of *Larix* charred needles in the peat layers of Fimon Lago core (FL, Fig. 1c) [23,220 ± 340 ^14^C years, 26,882–28,087 cal BP (2σ error)^48^] documents its presence in situ during local fires.

Similar mechanisms in fire activity are known in modern Canadian boreal forests, where bogs are more likely to burn in early season wildfires than other ecosystem types, even fire-prone upland conifers^113^. Further evidence supporting the large spatial scale nature of Fimon fires arises from the strong correlation between larger (local) charcoal particles and finer mesoscopic particles (i.e. 62–125 µm) (Fig. S5), which are a reliable proxy of wide burned areas (at least within 150 km)^114^. Similarly, results across western Canada suggest increasing peatland burned area during very large fires (> 140,000 ha) that likely occur during extreme fire conditions^115^. These very large fires can represent a significant amount of the total area burned on a regional scale, where typically 2–3% of all fires are responsible for 97% of the total area burned^116^.

### Linking HS 3 mid-latitude ecosystem variability to changes in circulation patterns and Greenland dust signals

Studies on ice-cores Ca^2+^ records, taken as dust proxy, display maxima in dust concentration during GS phases, particularly well-expressed during HS (Fig. 6a and Fig. S1). During GS 5.1 (HS 3) dust mainly sourced from central Asian deserts^11^, suggesting, as the main drivers of the observed dust increase, the pronounced continental aridity and increased soil dust availability coupled with more efficient atmospheric transport and reduced *en route* dust removal. Such reorganizations seem to have happened in close association to the millennial and-sub-millennial scale ITCZ-monsoon system variability^25–27^, as documented in low-latitudes high-resolution marine and terrestrial records. The GS 5.1 (HS 3) oscillation can be traced in great detail in the Cariaco Basin and Hulu cave records, where a more southerly annual position of the ITCZ^14,27^ and a weakening of summer East Asian Monsoon^86,117^ are inferred (Fig. 6b,c).

Although large uncertainties prevent any synchronisation of intra stadials/interstadials events between records, we note an intriguing signal modulation within the HS 3 interval in S-European records (Fig. 6d,e). The Lake Fimon record shows an absolute minimum in forest cover associated with increased concentration of long-distance windborne charcoal particles (10–50 μm sized) between 30,425 and 29,772 cal BP (2σ error) (event 2, Fig. 6e). It is followed by an intermediate step (event 3, Fig. 6e) preceding the abrupt start of Forest stage II (29,707–28,941 cal BP, 2σ error) at the onset of GI 4. This pattern resembles that of 7H speleothem that shows a peak of more depleted δ^18^O values at ca. 29.5–29.9 kBP^32^ (Fig. 6c), which is probably associated with a southern displacement of the storm track position and also correlated to peaks of major dust input in NGRIP record (Fig. 6a). The following intermediate step is consistent with a northward progression of the climate recovery towards interstadial conditions^32^. Interestingly, this sequence shares similarities with that found during GS9 (HS 4) in ice records, where synchronous changes in ^17^O-excess, δ^18^O_atm_, δD-CH4, methane and CO_2_, are interpreted as a three-phases fingerprint of the lower-latitude climate and hydrological cycle changes, most likely due to a southward shift of the ITCZ^118^. This is in agreement with recent data and modelling studies suggesting that the iceberg discharge only occurs several centuries after the cooling of ocean surface in North Atlantic and decrease of AMOC intensity^119,120^.

## Concluding remarks

Our study provides a new centennial to sub-centennial mid-latitude terrestrial palaeoecological record covering ca. 3800 years at MIS 3–2 transition. The median time-resolution of 58 years allows to detect even rapid events chronologically constrained by an independent radiocarbon chronology. Between 30,904 and 30,088 (end of Fimon forest stage I) and 29,707–28,941 (start of Fimon forest stage II) cal BP (2σ errors) occurred a long millennial phase of major ecosystem transformation, if compared to the other stadial cycles, related to GS 5.1 (HS 3) interval. Mixed open boreal forests were largely replaced by open environments with *Artemisia*-dominated semideserts, steppe and meadow-steppe, including Gramineae and Asteroideae. Data point to a remarkable shift from the boreal forest climate towards drier and colder conditions, at the continental edge of the forest-steppe ecological gradient. A long-distance (regional scale) fire signal is detected during HS 3. Extensive fires occurred at the site every 400 years (mFRI) during the following stadial/interstadial cycles, i.e. close to GI 4 and GI 3 boundaries, suggesting the influence of high-frequency climate variability on fire regime.

Finally, palaeoecological data from HS 3 interval unveiled an internal variability suggesting a peak between 30,425 and 29,772 cal BP (2σ error) which matches more depleted δ^18^O values in alpine speleothems. We hypothesise that this signal, on land, may be attributed to the southward shift of the NH storm tracks and the associated delayed iceberg discharge events as documented during other HS at the Iberian margin.

## Methods

### Chronology

The Fimon chronology was developed for the peaty-gyttja interval (i.e. 19.90–19.405 m, LZ1-2—see Fig. S2). We rely on four ^14^C dates made on bulk samples from the Fimon peat interval: three were obtained from the Fimon PD core (19.885–19.405 m, LZ2; Fig. S2 and Table 1) and one from the Fimon TdA core (Fig. 1c and Table 1). The latter was computed in the Fimon PD age-depth model after litho-, bio- and chronostratigraphic correlation of the two sequences^48^. We calibrated all dates using the IntCal20 calibration curve^78^ within OxCal 4.4 software^77^ and then calculated the age model using Bayesian analyses in OxCal, based on the Markov chain Monte Carlo algorithm. We used standard codes and commands in OxCal, including P_Sequence. The Fimon peat unit consists mostly of plant remains of helophyte and hygrophilous herbs which belong to Cyperaceae family (sedge) and *Filipendula* sp and *Potentilla* sp. (Fig. S2), not affected by (sub)recent roots from a visual inspection. This unit was radiocarbon dated in other two cores from the Fimon basin (FL and TdA, Fig. 1c) yielding comparable ages on bulk and terrestrial macrofossils samples (i.e., FL core)^48^. ^14^C dates made on pollen concentrates and bulk samples from the lowermost minerogenic unit (19.885–20.40 m; Fig. S3) yielded younger ages than the uppermost peat unit, mostly being age reversals (Table 1 and Fig. S3). Such lithological unit may suffer from downward mobility of the younger humic (and fulvic) acid fractions from the uppermost peat profile as previously demonstrated for the interval beyond 30 cal kBP^47^. For these reasons ^14^C ages from this lithological unit were excluded from our modelling.

### Loss on ignition (LOI)

73 volumetric samples were taken between 19.39 and 19.93 m, weighted and progressively heated at 105 °C, 550 °C and 980 °C to estimate water, total organic matter + sulphides (TOM + s) and the siliceous + oxides contents (RES). Total organic carbon (TOC) and the carbonate fraction, which includes also both sulphides (s) and sulphates (s) (CaCO3 + ss), were determined stoichiometrically^121^.

### Pollen analysis

54 samples were analysed at 1-cm intervals and prepared using standard methods (including HF and acetolysis) after adding *Lycopodium* tablets for pollen concentration and influx estimations^122^ at the Lab. of Palynology and Palaeoecology of CNR-IGAG in Milan. Pollen identification was carried out at the lowest taxonomic level possible at 400×, 630× and 1000× magnifications under a Leica DM-LB light microscope, using atlases^123–126^ and the CNR reference collection. Pollen diagrams were drawn using Tilia ver. 2.4.41^127^ and Corel Draw X8 for further graphic elaborations. The pollen sum used for % calculations includes trees, shrubs, and all upland herbs. Aquatics and wetland species are excluded. A minimum pollen count of 482 ± 56 grains has been reached. Pollen zonation was obtained through constrained incremental sum of squares cluster analysis (Cavalli Sforza's chord distance as dissimilarity coefficient—CONISS^128^). Clustering was restricted to taxa whose pollen reached over 2%. The clusters are represented in the dendrogram (Fig. 2). Pollen-slide charcoal particles were recognized under light microscope at 400×. Black, completely opaque and angular fragments^129^ were identified as charcoal within the size class 10–50 μm length. A principal component analysis (PCA) was performed on the covariance matrix of Hellinger-transformed % selected data (terrestrial pollen taxa > 2%). Data standardization and ordination were carried out with the Vegan package^79^ in R environment^80^. This multivariate analysis was used to extract the main vegetation gradients by detecting links between taxa and samples.

### Macrocharcoal analysis

Three different size ranges (62–125 µm, 125–500 µm and > 500 µm) of sieved charcoal particles were separated in 108 sediment samples of approximately 2 cm^3^ at contiguous 0.5 cm intervals using standard sieving methods^130^. Samples were gently disaggregated in a solution of 10% sodium hexametaphosphate, (NaPO_3_)_6_, and 12.5% sodium hypochlorite (NaClO) for 24 h and sieved (62, 125, 500-μm mesh). The sieved fractions were counted on a gridded platform using a stereomicroscope. Macroscopic charcoal particles (> 125 μm) are assumed to record high severity fires within a few kilometers of the study site^130,131^. Finer charcoal particles (62–125 µm size) are more widely dispersed and represent a reliable proxy of extra-local fire activity (at least within 150 km) as supported by dispersal models^114^. We obtained series of charcoal concentrations (particles cm^−3^) converted, for the 19.405–19.90 m interval, into total charcoal accumulation rates (CHAR, particles cm^−2^ year^−1^) by multiplying these values by sediment accumulation rates (cm year^−1^) inferred from the age–depth model (Fig. 3). The CHAR record (particles > 125 µm) was then decomposed into background (C_back_) and peak component using the method implemented in CharAnalysis 0.9 software^81^. Peaks, which are positive deviations from the C_background_ represent input of charcoal as a result of local (< 1 km) fires^132^. The C_background_ component was determined using a moving mode robust to outliers with a 500 years window width. A Gaussian mixture model was used to identify threshold values for peak identification (0.95 percentile). The fire frequency (FF) is the total number of fires within a 1000-year window. Fire return interval (FRI) is the time between two adjacent fire events. A Signal to Noise Index (SNI) was used to evaluate the suitability of sediment-charcoal records for reconstructing local fires. The SNI compares the variability in the signal population, var (S), to the variability in the noise population, var (N): SNI = var(S)/var(S) + var(N). A SNI greater than 3 consistently identifies records appropriate for peak detection^133^.

## Supplementary information

Supplementary Information

## Data Availability

All data generated or analysed during this study are included in this published article and its “Supplementary Information” files.
