# Supplementary material for: The fast-acting “pulse” of Heinrich Stadial 3 in a mid-latitude boreal ecosystem
Source: Sci Rep. 2020 Oct 22;10:18031. doi: 10.1038/s41598-020-74905-0 (PMC7581741; doi:10.1038/s41598-020-74905-0)
Supplement: Supplementary file 1 — Supplementary Information [file 41598_2020_74905_MOESM1_ESM.pdf]

**SUPPLEMENTARY INFORMATION**

# The fast-acting "pulse" of Heinrich Stadial 3 in a mid-latitude boreal ecosystem

Federica Badino[1,2]*, Roberta Pini[2], Paolo Bertuletti[2,3], Cesare Ravazzi[2], Barbara Delmonte[3], Giovanni Monegato[4], Paula Reimer[5], Francesca Vallè[3], Simona Arrighi[1,6], Eugenio Bortolini[1], Carla Figus[1], Federico Lugli[1,7], Valter Maggi[3], Giulia Marciani[1,6], Davide Margaritora[3,8], Gregorio Oxilia[1], Matteo Romandini[1,8], Sara Silvestrini[1], Stefano Benazzi[1,9]

[1] Department of Cultural Heritage, University of Bologna, 48121 Ravenna, Italy

[2] Research Group on Vegetation, Climate and Human Stratigraphy, Lab. of Palynology and Palaeoecology, CNR - Institute of Environmental Geology and Geoengineering (IGAG), 20126 Milano, Italy

[3] Department of Environmental and Earth Sciences, University of Milano-Bicocca, 20126 Milano, Italy

[4] CNR - Institute of Geosciences and Earth Resources (IGG), 35131 Padova, Italy

[5] School of Natural and Built Environment, Queen's University Belfast, Belfast BT7 1NN, UK

[6] Dipartimento di Scienze Fisiche della Terra e dell'Ambiente, Università di Siena, 53100 Siena, Italy

[7] Dipartimento di Scienze Chimiche e Geologiche, Università di Modena e Reggio Emilia, 41125 Modena, Italy

[8] Dipartimento di Studi Umanistici, Sezione di Scienze Preistoriche e Antropologiche, Università di Ferrara, 44100 Ferrara, Italy

[9] Department of Human Evolution Max Planck Institute for Evolutionary Anthropology, 04103 Leipzig, Germany

* Corresponding author: Federica Badino federica.badino@unibo.it

University of Bologna,

Department of Cultural Heritage

48121 Ravenna, Italy

http://www.erc-success.eu/

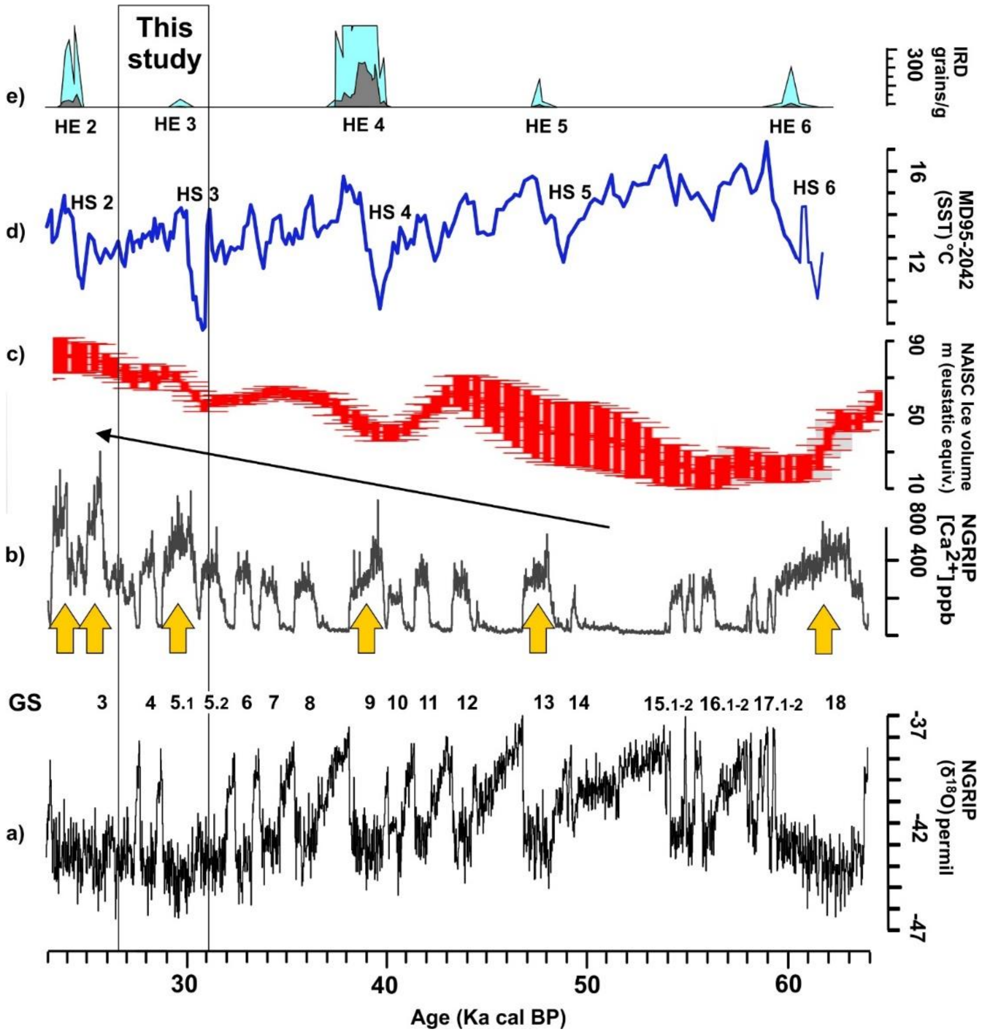


**Figure S1 –** a) NGRIP $\delta^{18}O$ record and b) calcium ion concentration ($[Ca^{2+}]$) record plotted on the GICC05modelext chronology[1]; c) Modelled ice sheet volume of the North American Ice Sheet Complex in eustatic equivalent metres of sea level (conversion factor of 25.19 m per 1015 $m^3$ of ice)[2]; d) MD95-2042 Sea Surface Temperature record[3] and (e) Ice-Rafted Debris record[4]. Record are plotted on their own timescale. Yellow arrows highlight phases of enhanced dust concentration corresponding to GS containing Heinrich Stadials.

## SI-1

### Geological and lithostratigraphic settings

The study area is located at the boundary between the western Veneto Plain, which is part of the foreland basin of the Southern Alps[5],[6] and the Berici Hils[7]. The Schio-Vicenza Fault System (SVFS, e.g., Pola et al., 2014 and references therein) is the tectonic structure separating these domains. The Berici Hills actually represented part of the Northern Apennine foreland[8] and are a tectonic slice unaffected by the Neoalpine tectonic phase[9],[10]. The Berici Hills are made up of

Eocene marls and marly limestone and calcarenite[11],[12] overlain by Oligocene shelf and lagoonal limestones[13].

The tectonic activity of the eastern Southern Alps led to an effective subsidence of the western Veneto Plain, in which the Quaternary successions, mostly terrestrial since the onset of Pleistocene glaciations, have been piled up[6],[14],[15]. This setting allowed the responsiveness of the plain to record major eustatic fluctuations and development of thick glaciofluvial bodies during glacial maxima.

Presently, the catchments of the Astico-Bacchigliona and Brenta rivers, to which the sedimentary succession of the western Veneto Plain belongs, developed in the Veneto Prealps and in the southern Dolomites. This catchment area includes different types of rocks, ranging from the Permo-Triassic and Jurassic-Cenozoic sedimentary successions as well the low-grade metamorphic basement, the Permian porphyries, Triassic volcanic rocks and the Palaeozoic plutonic rocks of Cima d'Asta[16],[17].

During the Late Pleistocene this sedimentary system has undergone depositional changes in response to drastic changing climatic and environmental conditions[18]. The Astico-Bacchiglione River likely flowed north of the Berici Hills during the Late Pleistocene before the LGM and the area of the outlet of Fimon valleys was underfed. The Brenta River was confined more eastward[19]. The first inlet of the Astico-Bacchiglione into the Fimon valleys took place at about 40 ka cal BP[20], while the Brenta River fed Lake Fimon soon after 26.5 ka cal BP at the onset of the megafan aggradation[19].

**SI-2**

**Depositional context and local environmental conditions at MIS 3-2 transition**

The Fimon PD core was sampled throughout a peaty-gyttja and clay interval ca. 0.5 m long (19.39-19.93 m depth). Lithofacies units were obtained by clustering LOI data in four main lithozones (LZ0-LZ3, **FIG.S2**). From bottom to the top: LZ0-Z1 are made of massive silty clay and slightly organic gyttja containing very high siliceous+ oxides values (90% of the dry weights) and very low Total Organic Matter + sulphides (TOM+s) contents (6-8%) up to 15% in LZ1. Then, maximum TOM+s contents (30-50%) between 19.885 and 19.405 m depth (LZ2) mark peat deposits mainly formed by thin layers of Cyperaceae leaves, which also yielded abundant pollen (**Fig. S2 and Fig.1**). The peat interval is attributed to long-lasting marshy conditions occupying a wide area (ca. 5.7 $km^2$) as recorded by the finding of the same peat layer, as documented by a detailed radiocarbon stratigraphy, in the three studied cores[20] and in other available stratigraphic data.

The general low and stable $CaCO_3$ content (2-3%) between 19.93 and 19.405 m (LZ0-1-2) suggests a negligible detrital carbonates input, which increases up to 5% from 19.40 m onwards (i.e., LZ3) in parallel with siliceous residue+ oxides increment (**Fig. S2**). This change, also marked by a sharp planktonic algae (*Pediastrum* and *Gloeotrichia*) peak (**Fig. S2**), points to a rapid lake-level rise driven by the external supply from the aggradation of the Brenta megafan (**Fig. 1b**) at the beginning of LGM in the southeastern Alps[19],[20].

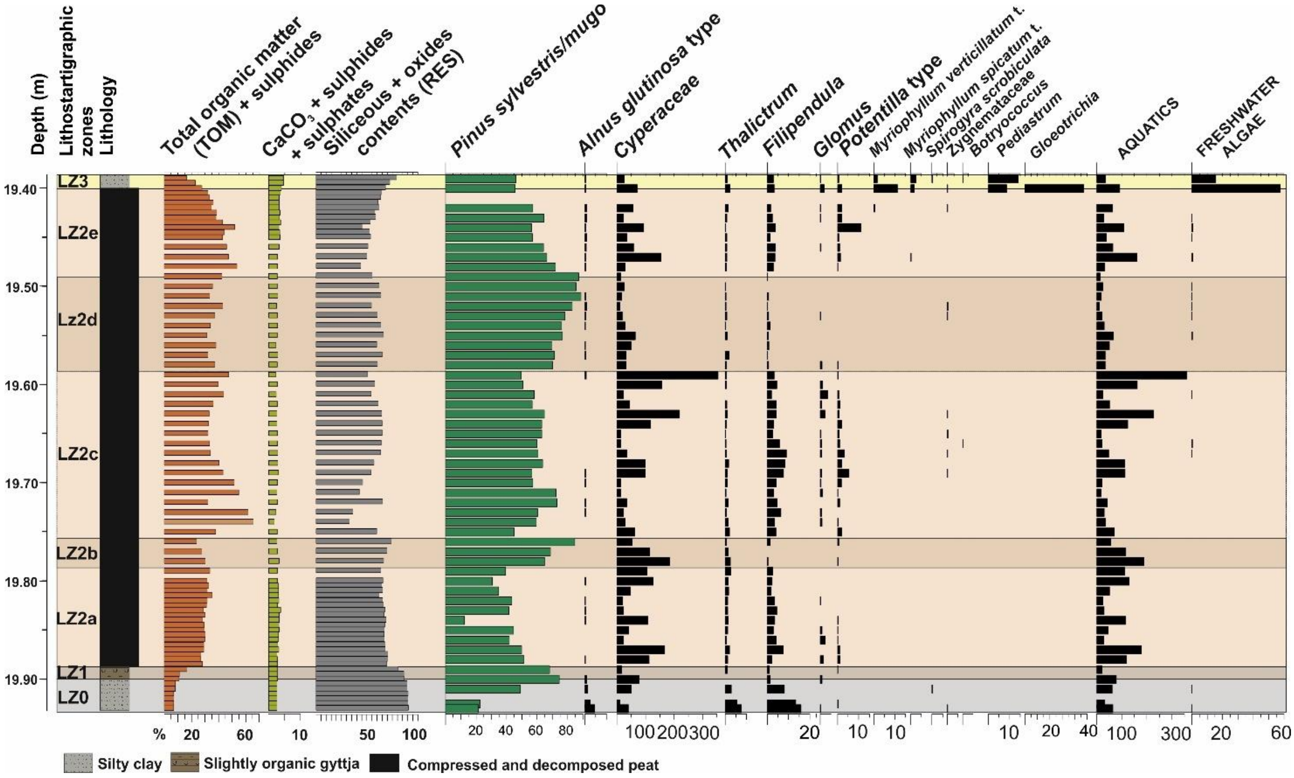


**Figure S2 –** Fimon PD depositional context and local environments at the MIS 3-2 transition.

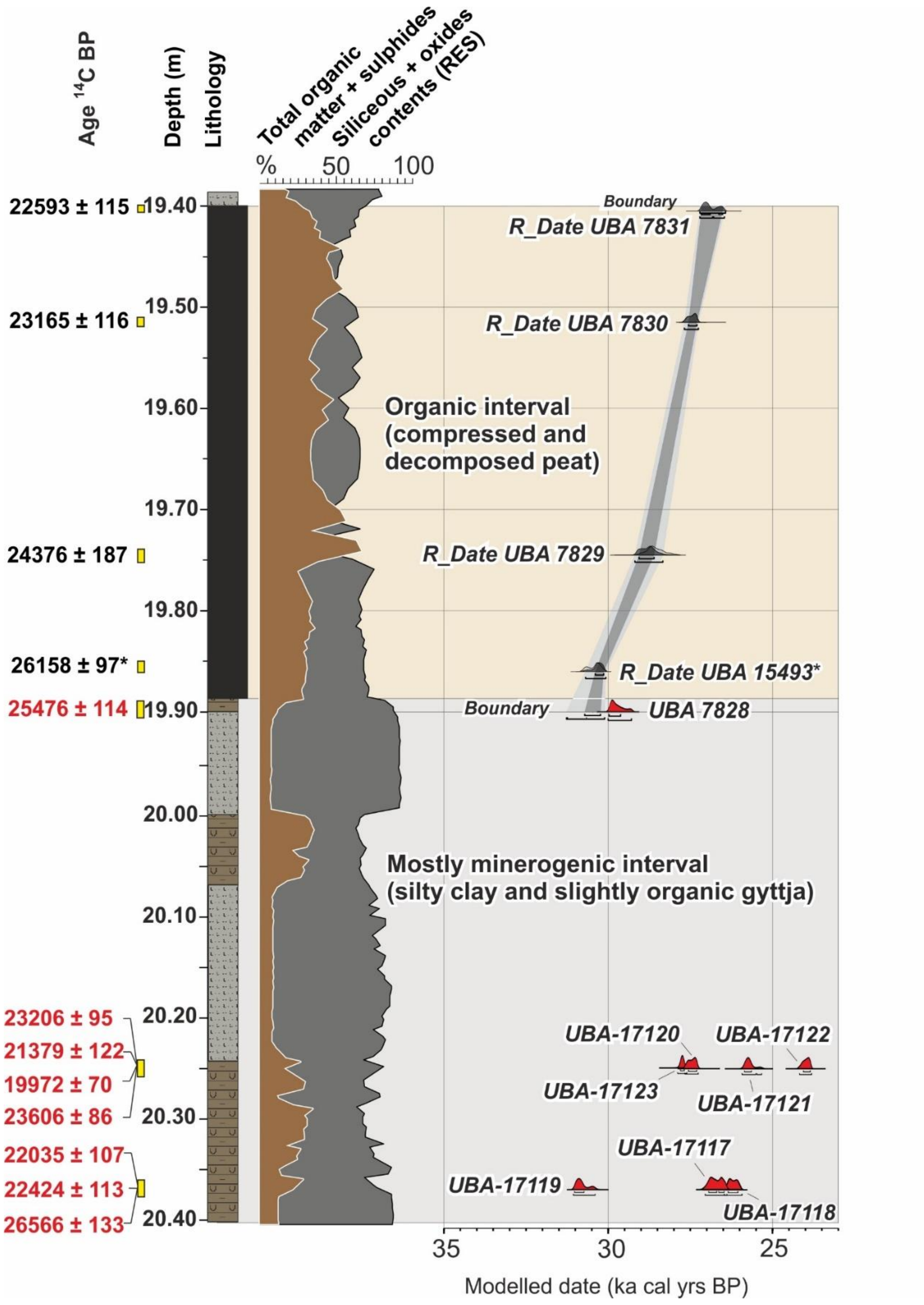


**Figure S3 –** Age-depth model of 19.90-19.405 m Fimon PD interval calculated with the OxCal 4.4 calibration software[21] using IntCal20 calibration curve[22]. The model relies on four $^{14}$C ages made on bulk samples from the peat unit (19.885 - 19.405 m, LZ2). * Indicates the $^{14}$C date

obtained from the Fimon TdA core (Fig. 1c) which was computed in the Fimon PD age-depth model after litho-, bio- and chronostratigraphic correlation of the two sequences[20]. $^{14}C$ ages made on pollen concentrates and bulk samples (see Table 1) from the lowermost minerogenic unit (19.885-20.40 m) yielded age reversals (see methods section for further details).

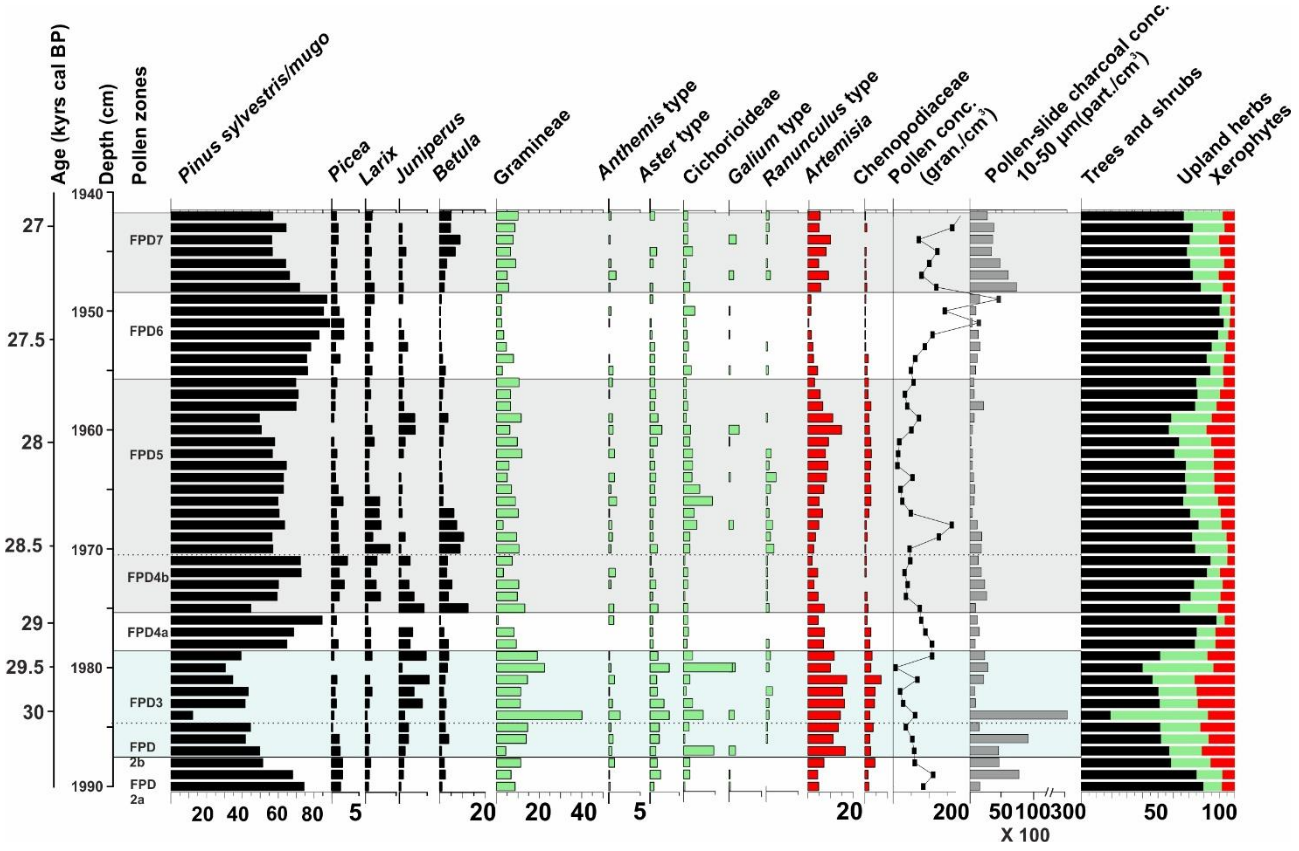


**Figure S4 -** Selected terrestrial percentage curves from the Fimon PD pollen record. Aquatics and wetland are excluded from the pollen sum. Light blue bar indicates a phase of major forest openness (FPD2b-3 pollen zone). Grey bars indicate FPD4b-5 and FPD 7 pollen zones.

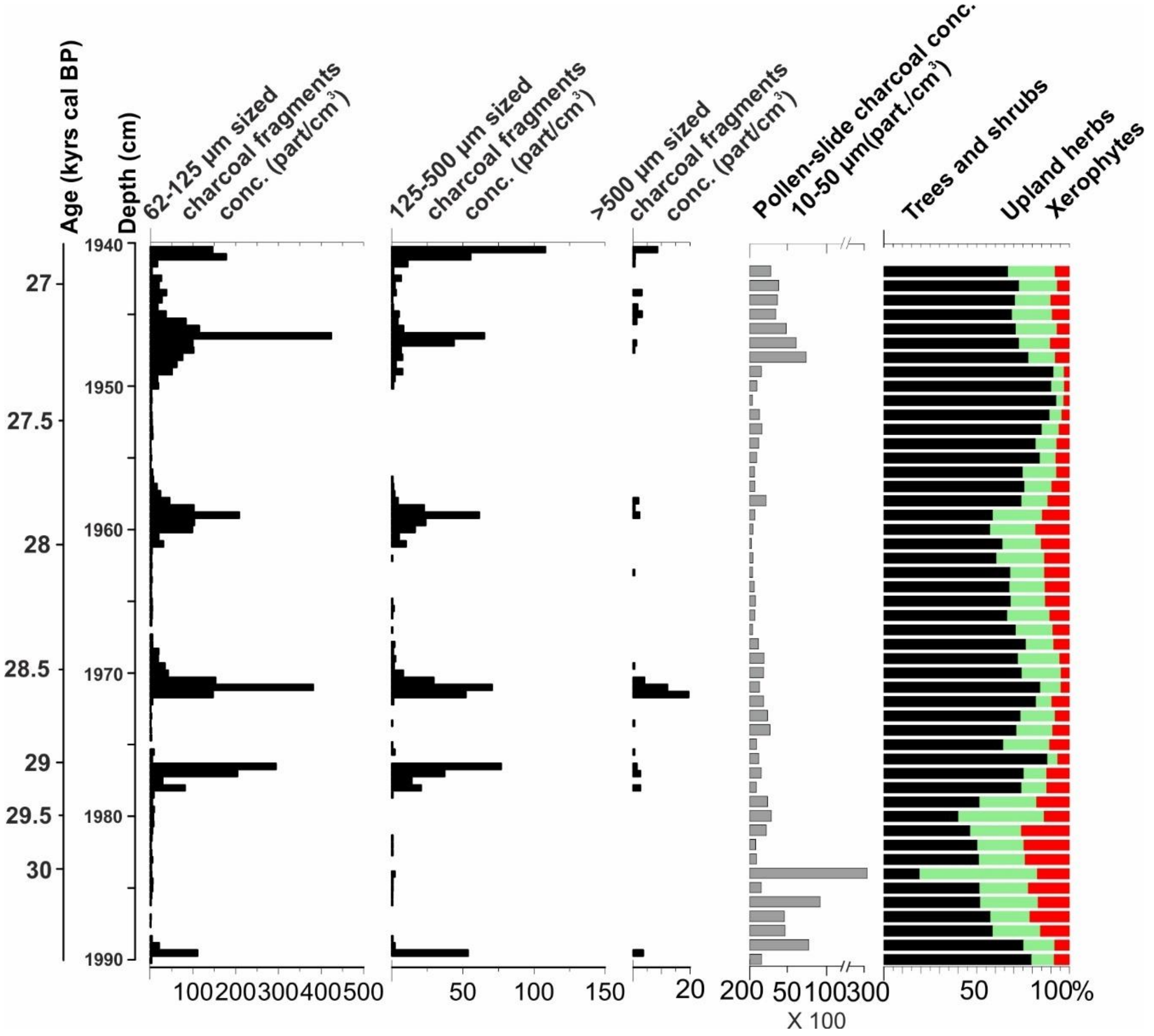


**Figure S5 –** Macrocharcoal and pollen-slide charcoal concentration records plotted against a synthetic diagram of terrestrial taxa: sum of trees and shrubs (black), sum of upland herbs (light green) and sum of xerophytes (red).

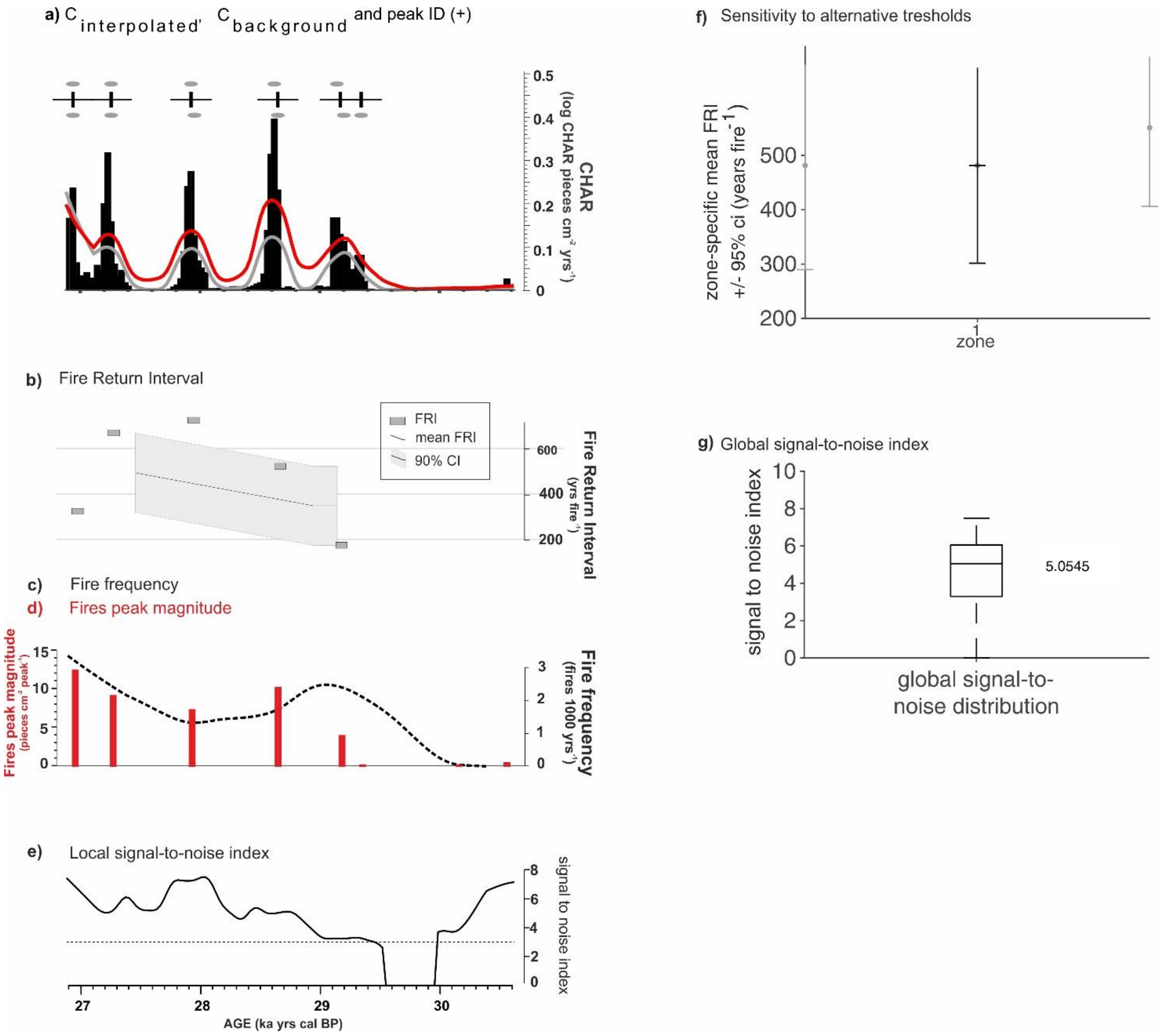


**Figure S6** - (a) Interpolated charcoal accumulation rate (pieces $cm^{-2}$ $yr^{-1}$) ($C_{interpolated}$= black histograms), low-frequency trend in CHAR, (pieces $cm^{-2}$ $yr^{-1}$) ($C_{background}$= grey) and Final positive CHAR threshold value (pieces $cm^{-2}$ $yr^{-1}$) (in red), with final peaks plotted as "+" symbols, and peaks1 and peaks2 plotted at gray dots; (b) Mean and raw Fire Return Intervals (FRI); c) Fire frequency (dashed black line) and d) Fires peak magnitude (red bars); e) The SNI for each sample, illustrating how the variability above Final positive CHAR threshold varies throughout a record; f) Mean fire return interval and 95% confidence limits (y-axis) for each zone (x-axis), based on (from left to right) peaks 1, peaks 2, and peaks 3. This illustrates the sensitivity of FRI interpretations to the three alternate thresholds entered in the input file; (g) Boxplot of all SNI values displayed in (e). Analysis were carried out using the method implemented in CharAnalysis 0.9 software[23].

| Fimon PD event stratigraphy | End of Forest stage I (cal yrs BP | Start of Forest stage II (cal yrs BP) | End of Forest stage II (cal yrs BP) | Start of Forest stage III (cal yrs BP) | End of Forest stage III (cal yrs BP) |
|---|---|---|---|---|---|
| Fimon PD palaeoecological record (radiocarbon based chronology) (This study) | 30904 – 30088 (2σ) | 29707 - 28941 (2σ) | 29250 - 28413 (2σ) | 27940 - 27443 (2σ) | 27550 - 27039 (2σ) |

| Other palaeoproxy records (non-tuned chronologies) | Start of GS 5.1/ HS 3 (cal yrs BP) | Start of GI 4 (cal yrs BP) | Start of GS 4 (cal yrs BP) | Start of GI 3 (cal yrs BP) | Start of GS 3 (cal yrs BP) |
|---|---|---|---|---|---|
| NGRIP $\delta^{18}O$ record (GICC05 chronology)[1] | 30550 ± 1008 (2σ) | 28850 ± 898 (2σ) | 28550 ± 887 (2σ) | 27730 ± 832 (2σ) | 27490 ± 822 (2σ) |
| 7H $\delta^{18}O$ record (U/Th chronology)[24] | / | 29059 ± 66 | 28,656 ± 64 | 27804 ± 54 | 27636 ± 65 |
| Sofular cave $\delta^{18}O$ record (U/Th chronology)[25] | 30200 | 29400 ± 29 | 29200 | 28000 ± 58 | 27700 |
| Hulu cave $\delta^{18}O$ record (U/Th chronology)[26],[27] | 30500 | 29347± 36 | / | 27988 ± 193 | / |

**Table S1**- Comparison of millennial and sub-millennial event boundaries as determined in Fimon PD record and their counterparts in other palaeoproxy records.

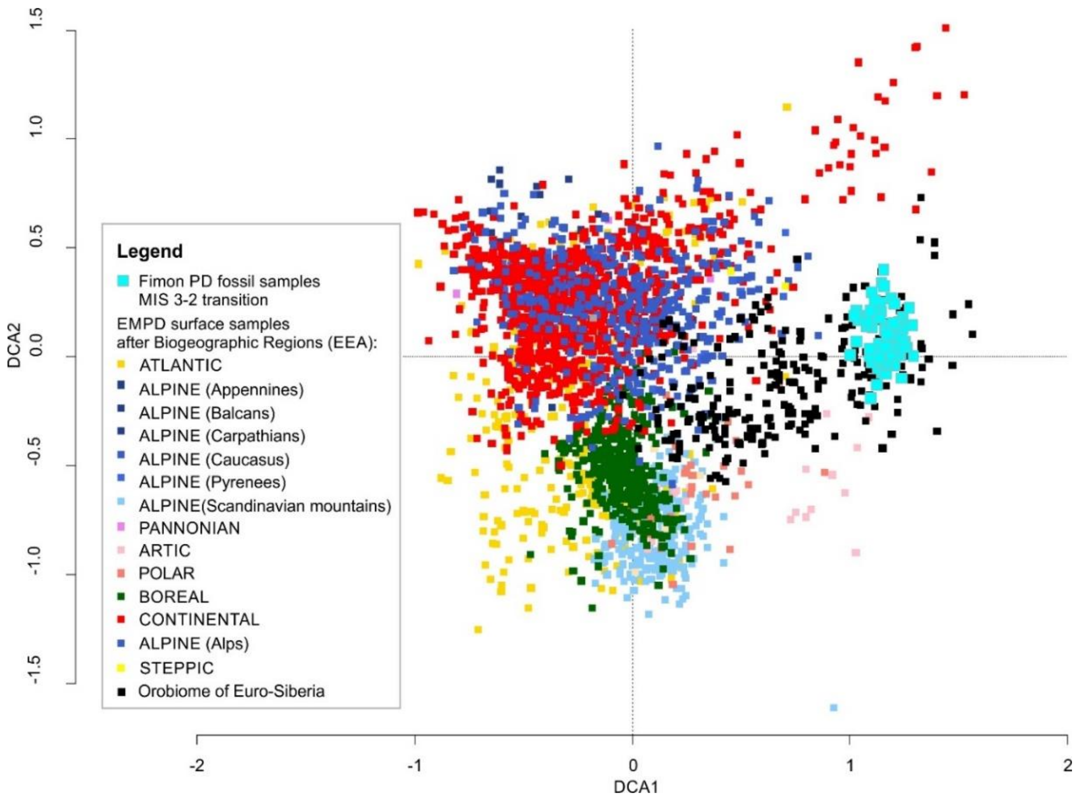


**Figure S7** – DCA biplot based on modern pollen samples (or assemblages) selected from European Modern Pollen database (EMPD)[28] across biogeographic regions (European Environmental Agency, https://www.eea.europa.eu/data-and-maps/data/biogeographical-regions-europe-3) and fossil pollen samples from Fimon PD site (bright light-blue). The closest modern analogues for Fimon LGM ecosystems appear to be the surface samples belong to the Euro-Siberian Orobiome (black) in the right part of the biplot. Data standardization and ordination were carried out with the Vegan package[29] in R environment[30].

**References**


1. Rasmussen, S. O. *et al.* A stratigraphic framework for abrupt climatic changes during the Last Glacial period based on three synchronized Greenland ice-core records: Refining and extending the INTIMATE event stratigraphy. *Quat. Sci. Rev.* **106**, 14–28 (2014).
2. Stokes, C. R., Tarasov, L. & Dyke, A. S. Dynamics of the North American Ice Sheet Complex during its inception and build-up to the Last Glacial Maximum. *Quat. Sci. Rev.* **50**, 86–104 (2012).
3. Darfeuil, S. *et al.* Sea surface temperature reconstructions over the last 70 kyr off Portugal: Biomarker data and regional modeling. *Paleoceanography* **31**, 40–65 (2016).
4. Sánchez Goñi, M. F. *et al.* Contrasting impacts of Dansgaard-Oeschger events over a western European latitudinal transect modulated by orbital parameters. *Quat. Sci. Rev.* **27**, 1136–1151 (2008).
5. Massari, F., Grandesso, P., Stefani, C. & Zanferrari, A. The Oligo-Miocene Molasse of the Veneto-Friuli region, Southern Alps. *G. di Geol.* **48**, 235-255. (1986).
6. Mancin, N., Di Giulio, A. & Cobianchi, M. Tectonic vs. climate forcing in the Cenozoic sedimentary evolution of a foreland basin (Eastern Southalpine system, Italy). *Basin Res.* **21**, 799–823 (2009).
7. Fabiani, R. La regione dei Berici: Morfologia, idrografia e geologia e carta della permeabilità delle rocce. in *Uff. Idrograf. Magistrato Acque* vols 28–29 1–84 (1911).
8. Fantoni, R. & Franciosi, R. Tectono-sedimentary setting of the Po Plain and Adriatic foreland. in *Rendiconti Lincei* vol. 21 197–209 (Springer, 2010).
9. Castellarin, A., Vai, G. B. & Cantelli, L. The Alpine evolution of the Southern Alps around the Giudicarie faults: A Late Cretaceous to Early Eocene transfer zone. *Tectonophysics* **414**, 203–223 (2006).

10. Márton´, E., Zampieri, D., Kázmér, M., Dunkl, I. & Frisch, W. New Paleocene-Eocene paleomagnetic results from the foreland of the Southern Alps confirm decoupling of stable Adria from the African plate. *Tectonophysics* **504**, 89–99 (2011).

11. Luciani, V., Negri, A. & Bassi, D. The Bartonian-Priabonian transition in the Mossano section (Colli Berici, north-eastern Italy): A tentative correlation between calcareous plankton and shallow-water benthic zonations. *Geobios* **35**, 140–149 (2002).

12. Bassi, D., Nebelsick, J. H., Puga-Bernabéu, Á. & Luciani, V. Middle Eocene Nummulites and their offshore re-deposition: A case study from the Middle Eocene of the Venetian area, northeastern Italy. *Sediment. Geol.* **297**, 1–15 (2013).

13. Ungaro, S. L'Oligocene dei Colli Berici. (1978).

14. Massari, F. *et al.* The environment of Venice area in the past two million years. *Palaeogeogr. Palaeoclimatol. Palaeoecol.* **202**, 273–308 (2004).

15. Kent, D. V., Rio, D., Massari, F., Kukla, G. & Lanci, L. Emergence of Venice during the Pleistocene. *Quat. Sci. Rev.* **21**, 1719–1727 (2002).

16. Antonelli, R. & Fabbri, P. Analysis and comparison of some values of transmissivity, permeability and storage from the Euganean Thermal Basin. IAHS-AISH publication. **176**, 707–718 (1988).

17. Avanzini, M., Bargossi, G. M., Borsato, A. & Selli, L. Note Illustrative della Carta Geologica d'Italia alla scala 1: 50.000, foglio 060- Trento, ISPRA-Servizio Geologico d'Italia, Trento. (2010).

18. Rossato, S. *et al.* Late Quaternary glaciations and connections to the piedmont plain in the prealpine environment: The middle and lower Astico Valley (NE Italy). *Quat. Int.* **288**, 8–24 (2013).

19. Rossato, S., Carraro, A., Monegato, G., Mozzi, P. & Tateo, F. Glacial dynamics in pre-Alpine narrow valleys during the Last Glacial Maximum inferred by lowland fluvial records

(northeast Italy). *Earth Surf. Dynam* **6**, 809–828 (2018).

20. Monegato, G., Pini, R., Ravazzi, C., Reimer, P. J. & Wick, L. Correlating Alpine glaciation with Adriatic sea-level changes through lake and alluvial stratigraphy. *J. Quat. Sci.* **26**, 791–804 (2011).

21. Bronk Ramsey, C. Bayesian Analysis of Radiocarbon Dates. *Radiocarbon* **51**, 337–360 (2009).

22. Reimer, P. *et al.* The IntCal20 Northern Hemisphere radiocarbon age calibration curve (0-55 kcal BP). *Radiocarbon* 1–33 (2020).

23. Higuera, P. E., Brubaker, L. B., Anderson, P. M., Hu, F. S. & Brown, T. A. Vegetation mediated the impacts of postglacial climate change on fire regimes in the south-central Brooks Range, Alaska. *Ecol. Monogr.* **79**, 201–219 (2009).

24. Luetscher, M. *et al.* North Atlantic storm track changes during the Last Glacial Maximum recorded by Alpine speleothems. *Nat. Commun.* **6**, 27–32 (2015).

25. Fleitmann, D. *et al.* Timing and climatic impact of Greenland interstadials recorded in stalagmites from northern Turkey. *Geophys. Res. Lett.* **36**, 1–5 (2009).

26. Wang, Y. J. *et al.* A high-resolution absolute-dated late pleistocene monsoon record from Hulu Cave, China. *Science* **294**, 2345–2348 (2001).

27. Cheng, H. *et al.* Atmospheric 14C/12C changes during the last glacial period from hulu cave. *Science* **362**, 1293–1297 (2018).

28. Davis, B. A. S. *et al.* The European Modern Pollen Database (EMPD) project. *Veg. Hist. Archaeobot.* **22**, 521–530 (2013).

29. Oksanen, J. *et al.* Package ‘vegan’. *R Packag. version 3.4.0* (2019).

30. R Development Core Team. A Language and Environment for Statistical Computing. *R Foundation for Statistical Computing* https://www.R-project.org (2019).
